# Using Near-Infrared Spectroscopy to Resolve the Species, Gender, Age, and the Presence of Wolbachia Infection in Laboratory-Reared Drosophila

**DOI:** 10.1534/g3.112.003103

**Published:** 2012-09-01

**Authors:** Wen C. Aw, Floyd E. Dowell, J. William O. Ballard

**Affiliations:** * School of Biotechnology and Biomolecular Sciences, University of New South Wales, Sydney 2052, Australia, and; † Engineering and Wind Erosion Research Unit, Center for Grain and Animal Health Research, United States Department of Agriculture, Agricultural Research Service, Manhattan, Kansas 66502

**Keywords:** Arthropods, species identification, sex, population age structure, parasite

## Abstract

The aim of the study was to determine the accuracy of near-infrared spectroscopy (NIRS) in determining species, gender, age, and the presence of the common endosymbiont Wolbachia in laboratory-reared Drosophila. NIRS measures the absorption of light by organic molecules. Initially, a calibration model was developed for each study. An independent set with flies not involved in initial cross-validation was then used to validate the accuracy of each calibration model. Flies from the independent sets were correctly classified into *Drosophila melanogaster* and *Drosophila simulans* with 94% and 82% accuracy, respectively, whereas flies were successfully classified by gender with accuracy greater than 90%. In the age grading test, correlation plots of the actual and predicted age for males and females of *D. melanogaster* and *D. simulans* were shown to be overlapping between the adjacent age groups. It is, however, possible to predict the age of flies as less than 9 days of age with 62–88% accuracy and flies that are equal to or older than 9 days of age with 91–98% accuracy. Finally, we used NIRS to detect the presence of Wolbachia in flies. Flies from the independent sets were successfully identified as infected or not infected with Wolbachia with approximately 90% accuracy. These results suggest that NIRS has the potential to quantify the species, gender, and presence of Wolbachia in fly populations. However, additional optimization of the protocol may be necessary before the technique can reliably estimate fly age.

Near**-**infrared spectroscopy (NIRS) has the potential to detect the chemical “fingerprint” of a specific sample. It detects the stretching and bending of CH, NH, and OH functional groups caused by the light absorption of organic molecules from 350 nm to 2500 nm (Pasquini 2003; Shenk *et al.* 2001). An additional benefit of NIRS is that it is noninvasive (Pasquini 2003), which means that live biological samples as well as untreated samples can be included in subsequent studies after screening. NIRS has been extensively used in a wide variety of situations, including the determination of the fat content in salmon (Sollid and Solberg 1992), oyster condition (Brown 2011), identification of minerals in mines (Duke 1994; Frost *et al.* 2005) and determination of wheat quality (Dowell *et al.* 2006). In insects, species identification studies that used NIRS have also been successfully completed in termites (Aldrich *et al.* 2007), mosquitoes (Mayagaya *et al.* 2009), and beetles (Perez-Mendoza *et al.* 2004). The NIRS method also has been used to determine the age of house flies (Perez-Mendoza *et al.* 2002), beetles (Perez-Mendoza *et al.* 2004), and mosquitoes (Mayagaya *et al.* 2009; Sikulu *et al.* 2011). The goal of this study was to determine whether NIRS is an appropriate tool for determining the species, gender, age, and the presence/absence of the symbiont Wolbachia in reared Drosophila. This research has the potential to serve as the basis for investigating the age-structure of Drosophila field populations and may be useful for modeling the spread of Wolbachia in nature.

In this study, NIRS first was used to differentiate two Drosophila sibling species by determining their metabolomic profiles. We then determined the accuracy of NIRS in determining the gender of each species. *Drosophila melanogaster* and *Drosophila simulans* are cosmopolitan human commensal sibling species. Male *D. melanogaster* and *D. simulans* can be distinguished by the external genitalia, but females are more difficult to identify (Sturtevant 1920). The Drosophila model has greatly contributed to the development of a wide range of disciplines, including, but not limited to, modern genetics (Morgan 1914), understanding of human diseases (Reiter *et al.* 2001), ageing (Ballard 2005; Ballard *et al.* 2007; Katewa and Ballard 2007a,b), and host−symbiont interactions (Hoffmann *et al.* 1990; Turelli and Hoffmann 1991, 1995; Min and Benzer 1997; Turelli 2010).

We tested whether NIRS can accurately determine the age of laboratory reared flies. Effective modeling of species with overlapping generations should include accurate information on population age structure (Hancock *et al.* 2011). If a population can be partitioned into gender-specific age groups that differ in survival rate and/or reproductive success, then it is important to identify the proportion of each age group to accurately model the dynamics of the entire population. Based on the proportion of each age group, a full life-table analysis with survival rate and fecundity can then be developed (Detinova 1968; Rasgon and Scott 2003). Up until the turn of the century, most of the age-grading methods for insects were based on changes in the reproductive system, such as the changes in follicular development, the appearance of the male and female reproductive tract, as well as the accumulation of follicular relics (Hayes and Wall 1999). Most of these techniques are destructive, inefficient, labor intensive, and require highly skilled analysis. In 2007, a new technique known as transcriptional age-grading was introduced. This method measured the variation of gene expression that changes with age and uses the resulting information to develop a transcriptional profile (Cook *et al.* 2007). Although this strategy is an important step forward, the technique is both expensive, time consuming, and destructive to the sample. As a consequence of these limitations, the NIRS method has been proposed as an important technical development for species identification of sibling species (Perez-Mendoza *et al.* 2002, 2004; Aldrich *et al.* 2007; Mayagaya *et al.* 2009; Sikulu *et al.* 2011) as well as age grading of the species (Perez-Mendoza *et al.* 2002, 2004; Mayagaya *et al.* 2009).

Finally, we determine whether NIRS can detect the presence of the common arthropod symbiont Wolbachia. Wolbachia is believed to infect at least 22% of all insects, including multiple species of Drosophila (Jeyaprakash and Hoy 2000; Werren and Windsor 2000). Although infection by Wolbachia is most usually maternally transmitted and primary affects the germ line cell, the symbiont frequently is found in nonreproductive host tissues (Dobson *et al.* 1999). In Drosophila, Wolbachia may cause cytoplasmic incompatibility, which results in sperm and eggs being unable to form viable offspring (Werren 1997). This phenomenon increases the fitness of Wolbachia-infected females and may drive the infection to spread rapidly within and between the populations (Turelli and Hoffmann 1991, 1995). During the last decades, the frequency dynamics of Wolbachia have been extensively studied and modeled in Drosophila (Hoffmann *et al.* 1990, 1999; Turelli and Hoffmann 1991). Wolbachia also has been proposed as a novel-biocontrol agent (Cook *et al.* 2008; McMeniman *et al.* 2009) and the rapid determination of its frequency in populations will impact upon the success of these endeavors.

A four-step approach was used in this study. First, we tested whether NIRS can differentiate the sibling species *D. melanogaster* and *D. simulans*. Second, we used NIRS to distinguish flies by gender. Third, we quantified the accuracy of NIRS in determining the age of laboratory-reared flies. Finally, we quantified the accuracy of NIRS in detecting whether a fly is infected with Wolbachia. Species, gender, and the presence of Wolbachia were identified with high accuracy, but the ability to determine the age of individual flies of each species was more varied.

## Materials and Methods

### Flies and husbandry

For the species, gender and age studies, two lines of Drosophila, *D. melanogaster* and *D. simulans*, were included. For consistency in this study, we refer to the *D. melanogaster* lines as *D. melanogaster* Alstonville (Alst) and *D. melanogaster* Dahomey (Dah). The lines were constructed by chromosome replacement using balancers and differed only in their mitochondrial genomes. To summarize, chromosomes X, 2, and 3 from the wild-type lines were replaced with homozygous chromosomes from the *w^1118^* iso line (Clancy 2008). The *D. simulans* lines were collected and maintained as isofemales in Kenya (Ky) in 2011 and Hawaii (Hw) in 2009. before the commencement of the study all flies were visually examined to ensure no mixture of species (Sturtevant 1920). For the Wolbachia identification test, two lines of *D. simulans* were included. The flies were collected in Hawaii as isofemales in 2009 and named Hw2 and Hw152. The flies were identified as being infected with the Wolbachia *w*Ha strain (C. C. Correa and J. W. O. Ballard, unpublished data). The Wolbachia uninfected strains were created by treatment of larval food with 0.03% tetracycline in water. Antibiotic-treated flies were tested by polymerase chain reaction (PCR) to confirm they were not infected with Wolbachia (Hoffmann *et al.* 1986). To minimize the influence of tetracycline, flies were raised at low density for at least five generations before the commencement of all studies (Ballard and Melvin 2007).

Flies were reared at 23° in a relative humidity of 50% under a 12:12-hr light:dark cycle. The density of flies in bottles was strictly controlled for at least two generations before the experiment was performed (Hercus and Hoffmann 2000). Oviposition resources (solidified agar−based medium containing 4% agar and 10% molasses) were placed in cages containing flies for 1 day. The eggs were collected and washed from the oviposition resources with 3% bleach followed by distilled water (Clancy and Kennington 2001). Approximately 170 eggs were transferred by pipette onto fly food. For the species, gender, and age studies larvae were raised on instant food (Carolina Biological Supply Company, Burlington, NC), and adults were transferred to adult food consisting of sucrose, yeast, agar, nipagen, and distilled water. For the Wolbachia identification test, larvae were fed on semolina, yeast agar diet, and then transferred to adult food.

### Near-infrared spectroscopy

As a basis for future field studies, a four-step approach was used to determine the utility of NIRS to identify the species, gender, age, and the presence of Wolbachia in laboratory-reared flies. Initially, a calibration model was developed for each study. An independent test set with flies not involved in initial cross-validation was used to validate the accuracy of each calibration model.

Either fresh flies or flies stored in RNA*later* (Ambion, Inc., Austin, TX) were included. For scanning of fresh flies, flies were anesthetized with humidified CO_2_ for 1 hr immediately before the scan was performed. For the Wolbachia identification test, fresh flies were submerged in RNA*later* in 0.6 mL of microcentrifuge tube and scanned after 1 week. The RNA*later* preservation method was shown to give similar accuracy in comparison with scanning the fresh insects (Sikulu *et al.* 2011). Flies were dried on a paper towel for 1 min before the scanning.

The system set-up follows Mayagaya *et al.* (2009). For scanning, about 25 flies were placed on a spectralon plate (ASD Inc., Boulder, Colorado, CO) and one fly scanned at a time. The flies were placed 2 mm below a 3-mm diameter bifurcated fiber-optic reflectance probe which contained 33 illumination fibers and 4 collection fibers. Diameter of probe was shown to affect the classification studies; therefore, all scanning were done using a 3-mm diameter reflectance probe (Mayagaya *et al.* 2009). In all studies, the probe was focused on the head and thorax on the dorsal side of the flies. Spectra were collected with a portable LabSpec 5000 spectrometer (350-2500 nm; ASD Inc.), and 50 spectra were collected from each fly and stored as an average spectrum. All spectra were collected using either ASD software Indico Pro 6.0.4 (ASD Inc.) or RS^3^ Spectral Acquisition Software 6.0.10 (ASD Inc).

### NIRS species identification

The first step of our approach was to determine whether NIRS could be used as a fast and effective strategy to distinguish between *D. melanogaster* and *D. simulans*. One calibration model was generated using flies of each species and gender. Calibration models were developed using *D. melanogaster* Alst and *D. simulans* Hw with five different age cohorts (1, 9, 13, 17, and 25 days after eclosion). *D. melanogaster* Dah and *D. simulans* Ky flies from two different age cohorts (5 and 21 days after eclosion) were subsequently included in the calibration model to increase the accuracy of species classification. The remaining flies in *D. melanogaster* Dah (5 and 21 days after eclosion) and *D. simulans* Ky (13 days after eclosion) were used as the independent set to estimate the accuracy of species identification. Flies that were 5, 13, and 21 days old were chosen in the independent set because these ages lie between the range of the calibration model (1−25 days after eclosion) and are critically important for accuracy prediction.

Average and individual spectra were compared between species. For all spectra, the known species of fly was compared with that predicted from the NIRS scan. In contrast to the actual species, which is discrete, the result obtained from the NIRS scan is a continuous variable. The closer the predicted scan result is to the actual or known result the greater the accuracy of prediction. The spectra of *D. melanogaster* flies were assigned a value of 1, and *D. simulans* flies were assigned a value of 2. The value of 1.5 was considered as the cut-off point for species identification. Flies with a predicted value less than 1.5 were classified as *D. melanogaster*, whereas those with a predicted value equal to or greater than 1.5 were classified as *D. simulans*.

### NIRS gender classification

The second step of our approach was to determine whether NIRS could distinguish the gender of each species. For the gender classification test, two calibration models were generated using *D. melanogaster* Alst and *D. simulans* Hw flies. Approximately 45 flies of each species and gender were scanned in seven age cohorts (1, 5, 9, 13, 17, 21, and 25 days after eclosion). Flies of different age groups were scanned in order and on different days. *D. melanogaster* Dah and *D. simulans* Ky were used as the independent set. For the independent set, approximately about 45 flies from each species and gender were scanned in three different age groups (5, 13, and 21 days after eclosion). The accuracy of the independent test set was then estimated.

Average and individual spectra were compared between males and females. For all spectra, the known sex of fly was compared with that predicted from the NIRS scan. For all spectra, males were arbitrarily assigned with a value of 3 and females a value of 4. The value of 3.5 was considered as the cut-off point for gender identification. Flies with a predicted value less than 3.5 were classified as male, whereas those with a predicted value equal to or greater than 3.5 were classified as female.

### NIRS age grading

The third step was to determine whether NIRS could age grade flies of each species and sex. In the laboratory, male and female Drosophila can age at different rates (Fukui *et al.* 1993). Male *w^1118^* flies were found to have a longer lifespan in comparison with females. For the age grading test, four calibration models were generated for two lines of each species and sex. Males and females of *D. melanogaster* Alst and *D. simulans* Hw were used to develop the calibration model. About 45 flies from each line and sex were scanned in seven age cohorts (1, 5, 9, 13, 17, 21, and 25 days after eclosion). The independent test set was generated using *D. melanogaster* Dah and *D. simulans* Ky flies. For the independent test about 45 flies from each line and sex were scanned in three different age groups (5, 13, and 21 days after eclosion) and the age cohort estimated using the appropriate calibration model.

Flies were assigned into three age groups according to their predicted age. Flies having a value of less than 9, 9-18, and greater than 18 days of age were predicted to be young, middle-aged, and old, respectively. Flies less than 9 days of age were considered young because no decline of lifespan in *w^1118^* flies was detected. Age 9-18 days was categorized as middle-aged because the mortality rate for *w^1118^* flies start increasing after 9 days old (Clancy 2008). We labeled flies greater than 18 days of age as old. In nature, few Drosophila likely survive longer than 26 days so flies older than 18 days of age are typically in the final third of their lifespan (Robson *et al.* 2006).

### NIRS Wolbachia identification

Finally, we assessed whether NIRS can detect the presence of Wolbachia in males and females *D. simulans*. For the Wolbachia identification test, calibration models were developed using two groups of Wolbachia infected and noninfected *D. simulans* (Hw2 and Hw152, both uninfected and infected). Flies were scanned at 15 days of age. *D. simulans* is predicted to have a generation time of approximately 25 days in nature, and matings are predicted to include males that are older than 14 days of age (Turelli and Hoffmann 1995). Flies that were excluded from the calibration model were used as the independent test set to estimate the accuracy of Wolbachia identification.

Average and individual spectra were compared between infected and uninfected flies. For all spectra in this test, the Wolbachia infected flies were arbitrarily assigned a value of 5 and noninfected flies a value of 6. The value of 5.5 was considered as the cut-off point for Wolbachia infection. Flies with a predicted value of less than 5.5 were classified as infected, while those with a predicted value equal to or greater than 5.5 were classified as non-infected flies.

### Data analysis

All spectra with ASD format were converted into SPC format by the Asd to Spc convertor version 6 (ASD Inc.). During the conversion, the spectra were transformed into Log 1/*R*. All spectra were mean-centered before analysis (Martens and Naes 2001) and then analyzed with GRAMS IQ 9.1 (Thermo Galactic, Salem, NH). The spectra from the independent test set were predicted with GRAMS IQ 9.1.

The initial examination of the average spectra showed considerable overlap between species. As a consequence, the calibration model was developed using partial least square (PLS) regression including flies from different age cohorts using the leave-one-out cross validation method (Mark 2001; Cawley and Talbot 2003). In this method, one sample is omitted from the sample set, and the remaining samples are used to generate a calibration equation. The omitted sample is predicted using the equation and then returned to the set followed by removal of the next sample. The process is repeated until all samples in the sample set are included in developing the equation. This technique slightly overestimates the accuracy of the prediction (Mark 2001).

Factor loading plots were used to identify the areas that have importance in the model. In addition, these plots can be used to identify areas that contain noise, which would be detrimental to the model. Factor loading plots show that the background noise of the spectra increases outside the 500−2200 nm region. As a consequence, the region outside of 500−2200 nm was excluded before statistical analysis. The spectra were preprocessed using the Savitzky−Golay (SG) first derivative. This correction will bring the spectra to a common baseline by pulling out changes in the Y-axis and increases the signal-to-noise ratio (Savitzky and Golay 1964; Schrader 2008).

The point smoothing function with maximum accuracy in the independent test set was chosen. For the species-identification test, the spectra were processed using the SG first derivative with 45-point smoothing function. For both the age grading and Wolbachia identification tests, the spectra were processed using the SG first derivative with 35-point smoothing function. In the gender-classification model, a slight decrease in accuracy was found in the SG processed spectra. Therefore, the original spectra without SG processing were included. The number of factors in the PLS regression plot was determined by predicted residual error sum of squares plot, which estimates the error that would be encountered in measuring new samples of a similar type. The calibration model with minimum number of factors in the predicted residual error sum of squares and maximum classification accuracy in the independent test set was chosen.

Outlier spectra were determined using spectral residual plot. This plot is useful for identifying samples that are spectrally different than the rest of the samples. In the spectral residual plot, outlier samples were marked using Mahalanobis distance. In summary, Mahalanobis distance measures the distance based on a set of multivariant data. The spectra were marked as outlier when the point of data spread outside 3 Mahalanobis distance. A further description of Mahalanobis distance is given by Mark (2001).

## Results

### NIRS species identification

**Figure 1:**
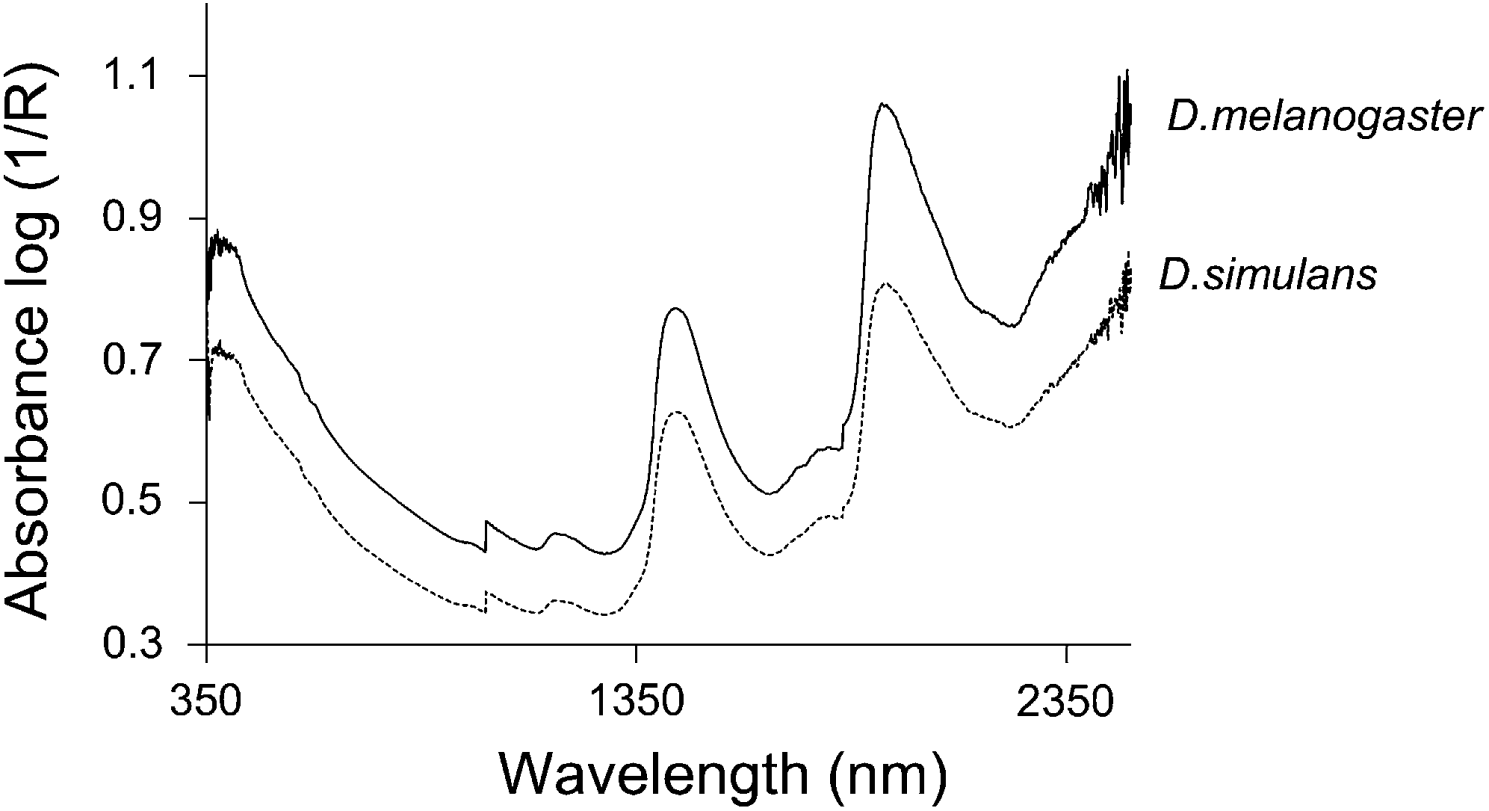
Mean spectra of all *D. melanogaster* and all *D. simulans samples*.

**Figure 2:**
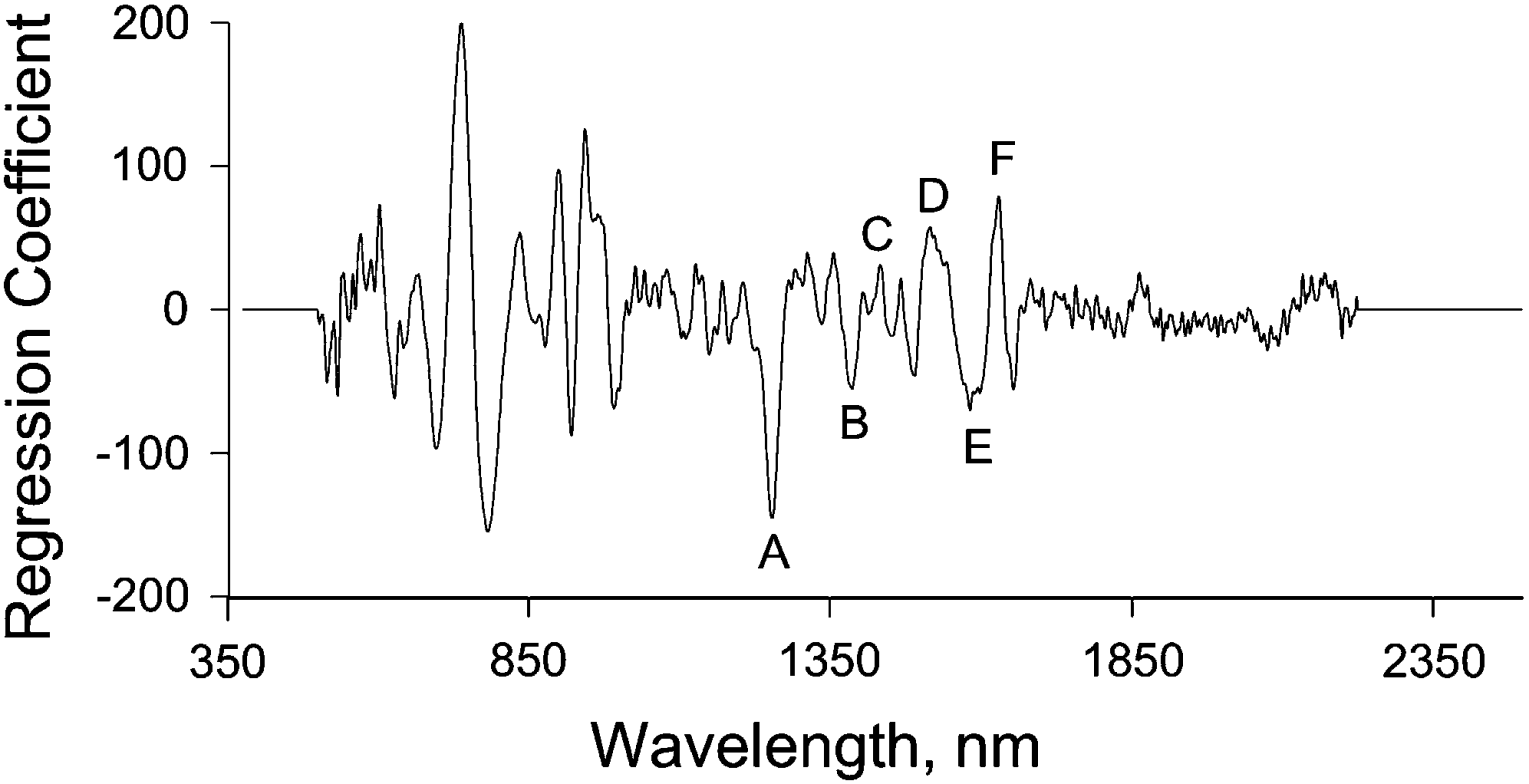
Regression coefficient plot for classifying *D. melanogaster* and *D. simulans* in the calibration model. The plot was generated with a 25-period moving average. (A) 1225 nm, (B) 1390 nm, (C) 1450 nm, (D) 1540 nm, (E) 1570 nm, (F) 1620 nm.

Initially, the individual and average spectra were examined visually. This initial analysis showed considerable overlap in individual spectra between species. Although there was noticeable offset in the average spectra, the pattern is broadly similar between the species (Figure 1). Plausibly, this offset may be caused by the physical effect of artifact light scattering, which does not carry any chemical or physical information. As a consequence, the PLS with cross-validation diagnostic method was used to develop the calibration model. A regression coefficient plot of the calibration model for species classification is shown in Figure 2. The regression coefficient displays the component of interest of the property being investigated and shows peaks in the region 500−1650 nm. Notably, peaks at regions around 1225, 1390, 1450, 1540, 1570, and 1620 nm show important NIR differences (Shenk *et al.* 2001) (Figure 2). Essentially these peaks show regions of differences between the species tested.

**Figure 3:**
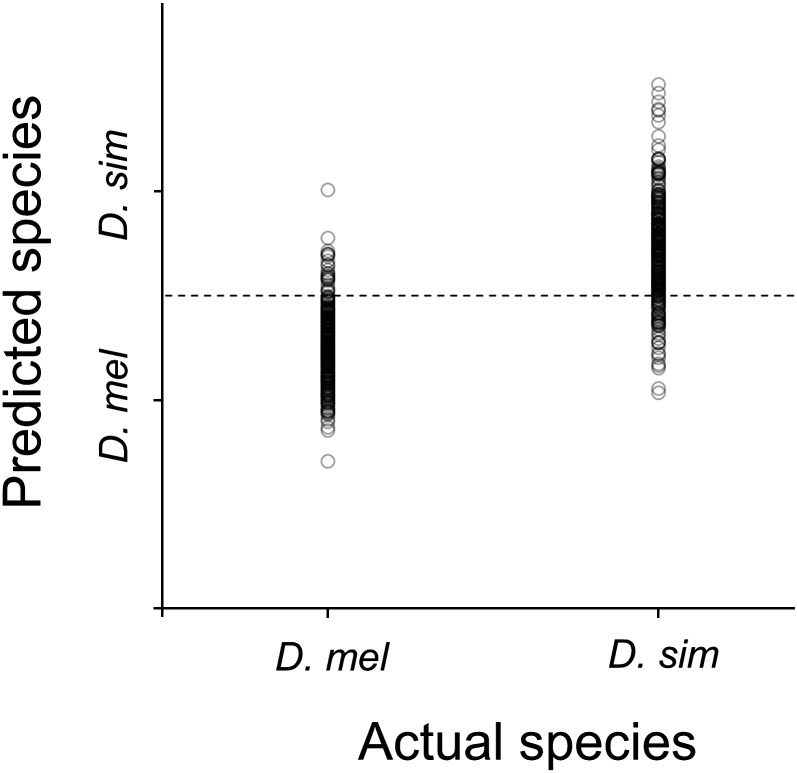
Near-infrared spectroscopy species prediction for *D. melanogaster* (*D. mel*) and *D. simulans (D. sim)*. Plot shows actual species against predicted species. Dotted line indicates cut-off point for delimiting species.

Flies from the independent set were correctly classified as *D. melanogaster* and *D. simulans* (Figure 3) with 94% and 82% accuracy, respectively (Table 1). *D. simulans* had a 8% reduction in accuracy of species prediction when the *D. simulans* Ky and *D. melanogaster* Dah lines were excluded from the calibration model. This reduction in accuracy was not obvious in *D. melanogaster* flies.

**Table 1:** Accuracy of *D. melanogaster* and *D. simulans* species identification in the independent set: The accuracy of prediction was compared using different calibration models

	Accuracy of Species Identification, %	
Calibration model	*D. melanogaster*	*D. simulans*
*D. melanogaster* Alst, n = 502	94	82
*D. melanogaster* Dah, n = 202		
*D. simulans* Hw, n = 472		
*D. simulans* Ky, n = 171		
*D. melanogaster* Alst, n = 705	93	74
*D. simulans* Hw, n = 649		

One calibration model was developed using two lines of *D. melanogaster* and *D. simulans*. Another calibration model with single lines of *D. melanogaster* and *D. simulans* was developed, and the accuracy of prediction was compared between both models.

### NIRS gender classification

**Figure 4:**
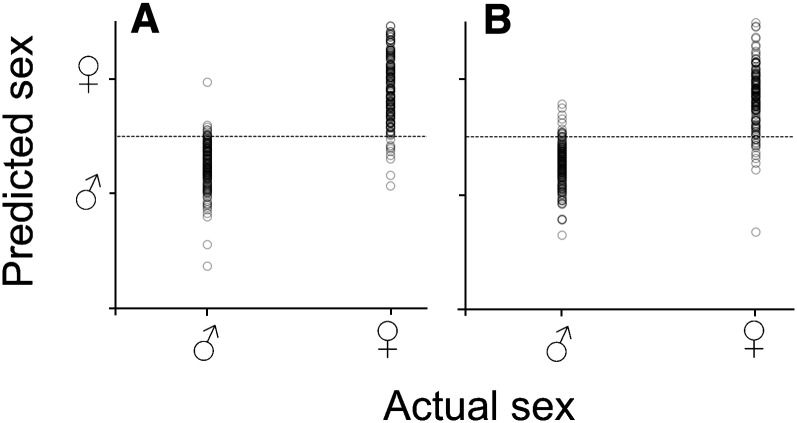
NIRS gender classification of Drosophila species. (A) Actual sex against predicted sex for male (♂) and female (♀) *D. melanogaster*. (B) Actual sex against predicted sex for male (♂) and female (♀) *D. simulans*. Dotted line indicates cut-off point for classification of sex.

Flies from the independent set were successfully classified by gender with accuracy greater than 90% (Table 2). Flies from the *D. melanogaster* Dah line were correctly classified as males and females with 95% and 97% accuracy, respectively (Table 2 and Figure 4A). For the *D. simulans* Ky line, the accuracy of gender classification was 94% in males and 92% in females (Table 2 and Figure 4B).

**Table 2:** Accuracy of *D. melanogaster* and *D. simulans* gender classification in the independent set

Accuracy of Gender Classification, %			
*D. melanogaster* Dah		*D. simulans* Ky	
Male, n = 152	Female, n = 172	Male, n = 158	Female, n = 153
95	97	94	92

### NIRS age grading

**Figure 5:**
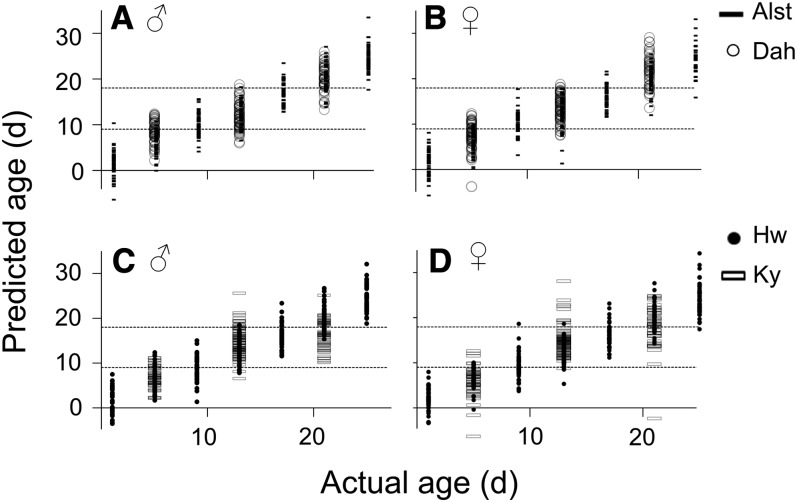
NIRS age grading of Drosophila species. Dotted lines refer to age classification of flies as either young (<9 days), middle-aged (9−18 days), or old (>18 days). Filled and nonfilled symbols represent values in the calibration model and independent set, respectively. (A) Actual *vs.* predicted age of male *D. melanogaster* Alst and Dah. Alst predicted age = 1.03 + (0.92 × actual age) (r^2^ = 0.88) (B) Actual *vs.* predicted age of female *D. melanogaster* Alst and Dah. Alst predicted age = 1.22 + (0.90 × actual age) (r^2^ = 0.86). (C) Actual *vs.* predicted age of male *D. simulans* Hw and Ky. Hw predicted age = 1.20 + (0.91 × actual age) (r^2^ = 0.87) (D) Actual *vs.* predicted age of female *D. simulans* Hw and Ky. Hw predicted age = 1.28 + (0.90 × actual age) (r^2^ = 0.87).

In the age-grading test, correlation plots of the actual and predicted age for males and females of *D. melanogaster* Alst and *D. simulans* Hw were shown to overlap between the adjacent age groups (Figure 5), Generally, the mean predicted age for males and females of both species is underpredicted for flies older than 13 days and overpredicted for flies younger than 9 days, with the exception of male *D. simulans* Hw (Table 3).

**Table 3:** Predicted age for males and females of *D. melanogaster* Alst and *D. simulans* Hw in the calibration model

	Predicted Age, Days							
	*D. melanogaster* Alst				*D. simulans* Hw			
	Male, n = 344		Female, n = 357		Male, n = 314		Female, n = 324	
Actual age, days	Mean	SD	Mean	SD	Mean	SD	Mean	SD
1	1.47	2.95	1.69	2.74	2.06	2.76	2.27	2.59
5	5.93	2.34	5.78	2.32	6.34	2.68	6.32	2.11
9	10.07	2.40	10.30	2.43	8.96	2.66	9.42	2.84
13	12.28	2.67	12.62	3.08	13.20	2.33	12.36	2.26
17	16.63	2.52	16.29	2.38	15.83	2.65	15.85	3.47
21	20.09	3.10	19.72	2.76	20.35	2.88	20.67	2.75
25	24.31	2.61	24.12	3.33	24.39	2.80	24.16	3.42

The independent set with different fly lines was developed to validate the accuracy of prediction of the calibration model. For the independent set, the *D. melanogaster* Dah line had a lower accuracy of age prediction in younger flies. In contrast, the *D. simulans* Ky line had a lower accuracy of age prediction in older flies (Table 4). Similar results were seen across both sexes. The accuracy of age prediction for flies in the independent set (< 9 days old, 9−18 days old, and >18 days) ranges from 37 to 88% (Table 4). The low accuracy of prediction was caused by the 21-day-old flies being predicted to be middle-aged (9−18 days old). It is, however, possible to distinguish *D. simulans* Ky flies as younger than 9 days of age, or older than, or equal to 9 days of age (Figure 5, C and D), with an accuracy of 78–98% (Table 4).

**Table 4:** Accuracy of age prediction of *D. melanogaster* Dah and *D. simulans* Ky in the independent set

Accuracy of Age Prediction, %				
Actual age, days	*D. melanogaster* Dah		*D. simulans* Ky	
	Male, n = 152	Female, n = 172	Male, n = 158	Female, n = 153
5	62	72	78	88
13	83	88	82	86
21	84	82	37	56
<9	62	72	78	88
≥9	91	95	95	98

### NIRS Wolbachia identification

**Figure 6:**
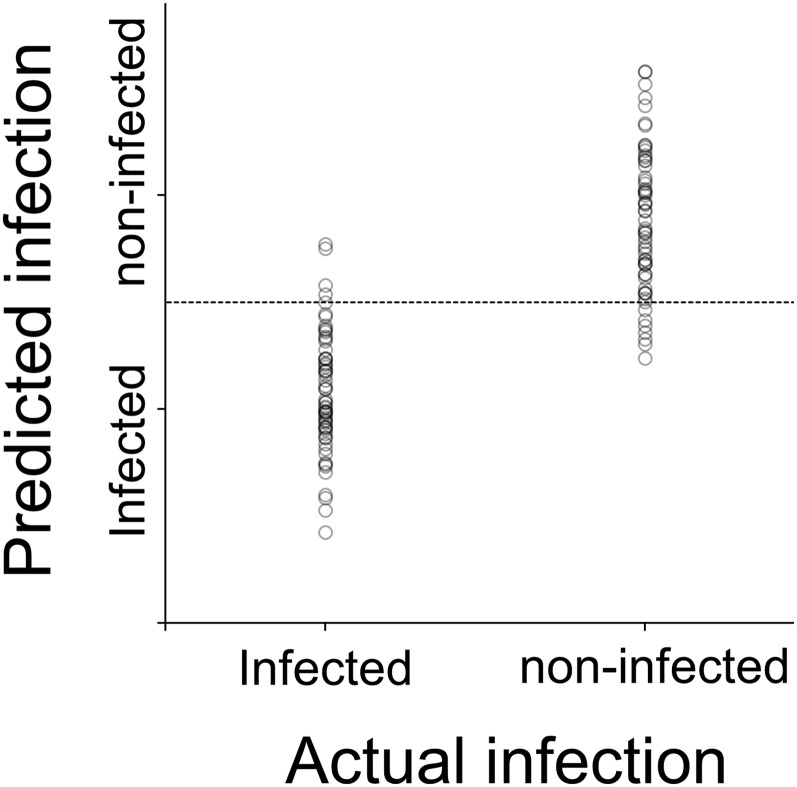
NIRS Wolbachia identification for Drosophila. Plot shows actual infection against predicted infection. Dotted line indicates cut off-point for delimiting infected and noninfected flies.

Finally, we used NIRS to detect the presence of Wolbachia in *D. simulans*. Flies from the independent test set were successfully identified as Wolbachia infected or noninfected with 87% and 92% accuracy, respectively (Table 5 and Figure 6).

**Table 5:** Accuracy of Wolbachia identification in the independent set

Calibration model N = 300	Independent set, n = 121	
	Infected	Noninfected
Infected Hw2	92%	87%
Infected Hw152		
Noninfected Hw2		
Noninfected Hw152		

## Discussion

NIRS is a noninvasive technique that detects absorption of light mainly by CH, NH, and OH functional groups from 350 to 2500 nm (Shenk *et al.* 2001; Pasquini 2003;). NIRS has been extensively used in a wide variety of situations, including cryptic species identification (Perez-Mendoza *et al.* 2004; Aldrich *et al.* 2007; Mayagaya *et al.* 2009). In this study, *D. melanogaster* and *D. simulans* were successfully identified with an accuracy of 80% or greater. Peaks displayed in regression coefficient plots represent differences between chemical compositions of the different Drosophila species included in this study (Figure 2). The functional group absorption in lipid and water contributed most of these peaks. Lipid absorption will give rise to the peak around region 1225, 1390, and 1620 nm, which is caused by the first overtones of CH group absorption as well as the combination of first, second, and third overtones of CH absorption in the CH2 and CH3 groups. Peaks located in the regions of 1450 and 1540 nm correspond to the first overtones of OH group absorption in water (Shenk *et al.* 2001). The observed differences in NIRS profiling are likely due to species-specific differences in lipid cuticle chemistry (Pennanec’H *et al.* 1995) that form a unique vibrational characteristic enabling species classification. In support of this hypothesis, studies have shown that female *D. melanogaster* and *D. simulans* have distinct cuticle hydrocarbons (Pennanec’H *et al.* 1995; Coyne 1996). In addition, variation in the amount of water content in flies may also play an important role in our species-classification test. Epicuticular lipids of arthropods serve as a barrier for evaporative water loss as well as playing roles in chemical communication between and within species (Gibbs *et al.* 1995, 1998). It was found that the length of the hydrocarbon chain, ratio of saturated to unsaturated hydrocarbons, as well as the methyl branching in the hydrocarbons have a direct influence on the rate of desiccation (Gibbs 1998). Variation in desiccation resistance has been found in Drosophila populations and species and this influences the rate of water loss and the amount of water content in adult flies (Hoffmann and Harshman 1999). In some strains of Drosophila at least, these differences in desiccation rate can be explained largely by the genetic variation in the expression of cuticle hydrocarbon among the flies and is likely to be important for climate adaptation and mate choice (Foley *et al.* 2007; Foley and Telonis-Scott 2011).

The gender of two Drosophila species was successfully predicted in our study. This finding is expected because the amounts of hydrocarbons differ between males and females in Drosophila (Jackson *et al.* 1981; Parisi *et al.* 2011). In comparison with males, females have a greater level of polyunsaturated cholesterol ester and a lower level of lysophosphatidylcholine (Parisi *et al.* 2011). Moreover, Drosophila females tend to be larger than males (Cowley and Atchley 1988; Gibbs and Markow 2001), which may be due to differences in lipid levels and composition required for egg production (Fairbanks and Burch 1970; Djawdan *et al.* 1996).

Age-grading of flies was predicted with lower accuracy than the three other variables tested, and additional optimization of the protocol will need to be considered before the technique can reliably estimate the age of field collected flies. Most generally, the mean predicted age for males and females of both species is underpredicted for flies older than 13 days and overpredicted for flies younger than 9 days. In *D. melanogaster*, the Alst line was used to develop a calibration model to predict the independent test set of Dah line (Figure 5). A higher accuracy of age estimation for *D. melanogaster* compared with *D. simulans* is expected because the lines used in developing the calibration model and independent test set have a homogeneous nuclear background (although some variation may exist on chromosome four) and the mitochondrial DNA differs by just two amino acids (Clancy 2008). In comparison, the *D. simulans* flies were collected from geographically distant locations. *D. simulans* could be classified as young (less than 9 days old) or as middle-aged/old flies (≥9 days of age). It has long been known that the lipid cuticle hydrocarbon content changes with age in house flies. Notably, the level of unsaturated hydrocarbon is much greater in older houseflies (Gibbs *et al.* 1995). The same hydrocarbon changes also were observed in Drosophila species. In *D. melanogaster*, the level of 7,11-nonacosadiene was found to increase with age. In contrast, 7,11-hexacosadiene decreases with age (Kuo *et al.* 2012). Based on the aforementioned findings, we hypothesize that the age-grading model in our study detected the age-specific changes in cuticle hydrocarbon. NIRS has been successfully used in age grading of houseflies and mosquitoes. In house flies, 93% of flies were correctly classified into two groups: younger than or equal to 3 days of age, or older than or equal to 7 days of age (Perez-Mendoza *et al.* 2002). In mosquitoes, approximately 80% of female mosquitoes were correctly classified as either younger than 7 days of age or older than or equal to 7 days of age (Mayagaya *et al.* 2009).

Our study shows the Wolbachia *w*Ha-infected Drosophila can be successfully detected using NIRS. Wolbachia are small intracellular bacteria with density varying between tissues and strains (Ijichi *et al.* 2002). One possibility is that NIRS is measuring the Wolbachia load in the flies. Wolbachia are Gram-negative bacteria that contain lipopolysaccharide molecules (Zhang *et al.* 2000). Lipopolysaccharides consist of lipid A fatty acids and polysaccharide linked with three covalent bonds (Morrison and Ryan 1992). If the amount of lipopolysaccharide molecules is high enough, these lipid and polysaccharide compounds containing CH and OH groups may be detected by NIRS. An alternative hypothesis is that the NIRS is detecting the changes of physiological effects on Wolbachia-infected flies. For example, it has been hypothesized that Wolbachia may obtain cholesterol from the host to synthesize lipopolysaccharide molecules (Lin and Rikihisa 2003; Brownlie and O’Neill 2006) and this may be detected by NIRS. Correlating Wolbachia density or cholesterol level in flies with NIRS measurements would facilitate resolution of these alternate hypotheses. Additional studies, including flies that are infected with different strains of Wolbachia, will be required for a more detailed understanding of the functionality of NIRS in Wolbachia detection.

NIRS has specific strengths and weaknesses when compared to more traditional diagnostic methods such as PCR. PCR can be highly accurate but tends to be relatively expensive and time-consuming, which tends to limit its application to smaller sample sizes. In comparison, running costs are low after initial investment of the NIRS instrument, and more than 1000 samples can be analyzed by NIRS in 1 day (Mayagaya *et al.* 2009). An additional benefit of NIRS scanning is that it is nondestructive (Pasquini 2003) so subsequent morphologic or biochemical studies can be performed on each sample after the scanning. However, NIRS has a lower accuracy of prediction than PCR. There are at least three approaches that can be considered to improve the accuracy of prediction by NIRS. The simplest method is to increase the sample size of the calibration model. A second possibility is to include a broader range of wavelengths. Unfortunately, the current combined mid-IR/near-IR spectroscopy is not portable and therefore not suitable for field work. A third more complex alternative may be to extract and measure the constituents of interest rather than measuring the whole sample. For example, if the cuticle lipid in this study is the main constituent of measurement, extracting and scanning only the cuticle lipid may help to reduce noise generated by the interference of other components. A disadvantage of this latter alternative is that the fly may be destructively sampled.

A subsequent challenge is to test whether this strategy is also applicable to wild flies. There are at least two factors that have to be considered to accurately estimate flies collected from the field. First, the development of Drosophila is temperature dependent (Miquel *et al.* 1976; Angilletta *et al.* 2004). This study was conducted under controlled laboratory conditions, and the changes in environmental temperature may lead to different results. Therefore, the age-prediction calibration model should be related back to the environmental temperature where the flies were collected. Second, wild-caught flies are more genetically variable and not inbred as those included in this study. A consequence of the genetic variation is that calibration models should be developed from flies collected in each experimental location. The influence of genetic variation on age grading was clearly demonstrated in this study and it is expected to affect the accuracy of prediction of wild flies.
